# Activation of Notch3 in Renal Tubular Cells Leads to Progressive Cystic Kidney Disease

**DOI:** 10.3390/ijms23020884

**Published:** 2022-01-14

**Authors:** Sonja Djudjaj, Panagiotis Kavvadas, Niki Prakoura, Roman D. Bülow, Tiffany Migeon, Sandrine Placier, Christos E. Chadjichristos, Peter Boor, Christos Chatziantoniou

**Affiliations:** 1 Institute of Pathology, University Hospital RWTH Aachen, 52074 Aachen, Germany; sdjudjaj@ukaachen.de (S.D.); rbuelow@ukaachen.de (R.D.B.); pboor@ukaachen.de (P.B.); 2 Unite Mixte de Recherche Scientific 1155, Institut National de la Sante et de la Recherche Medicale, Tenon Hospital, 75020 Paris, France; kavvadasp@gmail.com (P.K.); niki.prakoura@gmail.com (N.P.); sandrine.placier@aphp.fr (S.P.); christos.chadjichristos@upmc.fr (C.E.C.); 3 Faculty of Medicine, Sorbonne University, 75020 Paris, France; tiffany.migeon@upmc.fr; 4 Division of Nephrology and Clinical Immunology, University Hospital RWTH Aachen, 52074 Aachen, Germany

**Keywords:** Notch3, polycystic kidney disease, renal cell carcinoma, renal fibrosis, chronic kidney disease, renal inflammation

## Abstract

Background: Polycystic kidney disease (PKD) is a genetic disorder affecting millions of people worldwide that is characterized by fluid-filled cysts and leads to end-stage renal disease (ESRD). The hallmarks of PKD are proliferation and dedifferentiation of tubular epithelial cells, cellular processes known to be regulated by Notch signaling. Methods: We found increased Notch3 expression in human PKD and renal cell carcinoma biopsies. To obtain insight into the underlying mechanisms and the functional consequences of this abnormal expression, we developed a transgenic mouse model with conditional overexpression of the intracellular Notch3 (ICN3) domain specifically in renal tubules. We evaluated the alterations in renal function (creatininemia, BUN) and structure (cysts, fibrosis, inflammation) and measured the expression of several genes involved in Notch signaling and the mechanisms of inflammation, proliferation, dedifferentiation, fibrosis, injury, apoptosis and regeneration. Results: After one month of ICN3 overexpression, kidneys were larger with tubules grossly enlarged in diameter, with cell hypertrophy and hyperplasia, exclusively in the outer stripe of the outer medulla. After three months, mice developed numerous cysts in proximal and distal tubules. The cysts had variable sizes and were lined with a single- or multilayered, flattened, cuboid or columnar epithelium. This resulted in epithelial hyperplasia, which was observed as protrusions into the cystic lumen in some of the renal cysts. The pre-cystic and cystic epithelium showed increased expression of cytoskeletal filaments and markers of epithelial injury and dedifferentiation. Additionally, the epithelium showed increased proliferation with an aberrant orientation of the mitotic spindle. These phenotypic tubular alterations led to progressive interstitial inflammation and fibrosis. Conclusions: In summary, Notch3 signaling promoted tubular cell proliferation, the alignment of cell division, dedifferentiation and hyperplasia, leading to cystic kidney diseases and pre-neoplastic lesions.

## 1. Introduction

Cystic kidney diseases are characterized by fluid-filled cysts, which compress the surrounding renal parenchyma and derogate renal function [1]. They occur due to genetic mutations or can be acquired over time in patients with chronic kidney disease. Formation of renal cysts can begin as early as during fetal development and progress to end-stage renal diseases during the midlife years of a patient [2]. Cystic kidney disease is characterized by ciliary dysfunction, epithelial hyperplasia lesions, basement membrane abnormalities, such as mislocalization of NaK-ATPase, and tubulointerstitial inflammation and fibrosis [1,2,3].

The Notch receptor family consists of four members (Notch1–4) and is an evolutionarily conserved intercellular signaling pathway involved in numerous biological processes including cell fate determination, cellular differentiation, proliferation, survival and apoptosis [4]. Notch ligands, belonging to the Delta and Jagged families, bind to the receptor and activate a cascade of proteolytic cleavage processes that release the Notch intracellular domain (NICD). The NICD translocates to the nucleus, binds the transcription regulator Rbpj and activates transcription of its downstream targets of the HES (Hairy and enhancer of split) and HEY (Hairy/enhancer-of-split related with YRPW motif) (or HRT—Hairy-related transcription factor) families [5].

Notch3 is expressed by vascular smooth muscle cells and regulates vascular development and reactivity [6]. We have previously demonstrated that in pathologic conditions, Notch3 is ectopically expressed by injured cells and promotes renal disease [7,8,9]. In chronic and acute kidney injury models, kidney tubular epithelial cells expressed Notch3 de novo, which mediated tubular proliferation and pro-inflammatory responses. Activation of Notch3 signaling in renal epithelial cells dramatically deteriorated the cell phenotype after injury, while Notch3 inhibition rescued renal function and structure [9]. Clinical and experimental data suggest a role of Notch signaling in renal cystogenesis. Mutations in JAG-1 and Notch-2 cause Alagille syndrome (AGS). Affected children have a deficiency in intrahepatic bile ducts, facial and skeletal anomalies and renal manifestations. While renal dysplasia accounts for most of the renal manifestations, bilateral renal cysts have also been identified in patients with AGS [10].

In this study, we examined whether Notch3 is involved in renal epithelial cyst formation.

## 2. Results

### 2.1. Notch3 Is De Novo Expressed in Epithelial Cells in Renal Carcinoma and Polycystic Kidney Disease

**Figure 1:**
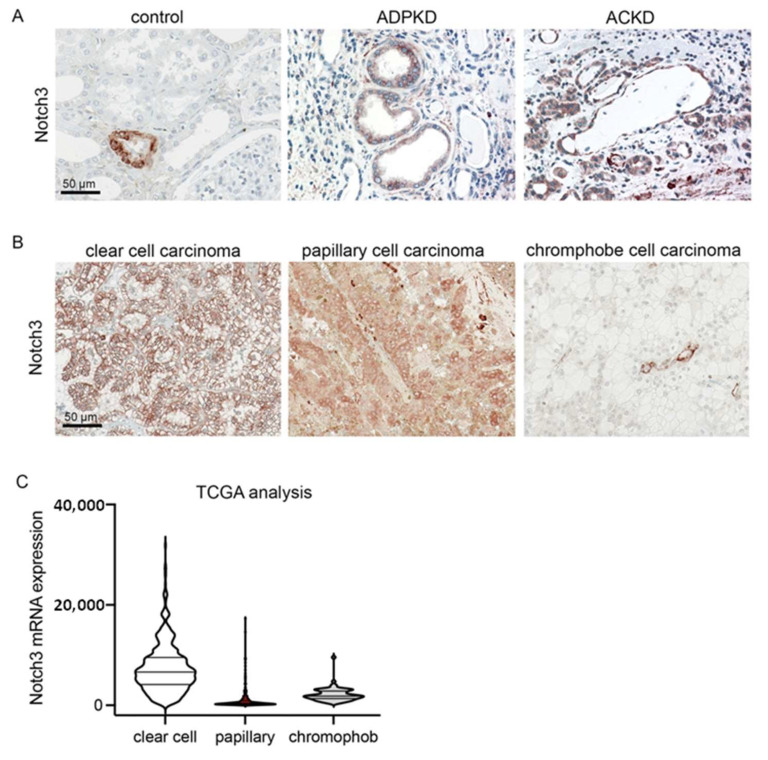
Notch3 expression is increased in polycystic kidney disease and renal cell carcinoma (RCC). Immunohistochemical staining for Notch3 in healthy control renal tissue and in tissue of patients with autosomal dominant polycystic kidney disease (ADPKD) and acquired cystic kidney disease (ACKD) demonstrated a de novo expression of Notch3 in dilated tubules in ADPKD as well as ACKD (**A**). Immunohistochemical staining of biopsies from patients with different types of renal cell carcinoma also showed increased Notch3 expression (**B**). Reanalysis of publicly available TCGA confirmed increased Notch3 expression in renal cell carcinoma (**C**).

To examine whether Notch3 plays a role in polycystic disease in humans, we analyzed the expression of Notch3 in human biopsies from patients suffering from autosomal dominant polycystic kidney disease (ADPKD) and acquired cystic kidney disease (ACKD). Notch3 was highly upregulated in all patients with ADPKD (n = 5) and ACKD (n = 5). It was mainly expressed by tubular epithelial cells lining the cysts (Figure 1A). Tumor-distant nephrectomy tissue from tumor nephrectomies without another renal disease served as a control. Here, Notch3 was only expressed by α-smooth muscle cells in the vessels.

We further analyzed human biopsies from patients with different subtypes of renal cell carcinoma (RCC). In all cases of RCCs, Notch3 was expressed by tubular epithelial cells (Figure 1B). Reanalysis of publicly available data from The Cancer Genome Atlas (TCGA) revealed that *NOTCH3* mRNA was expressed most strongly in clear cell renal cell carcinomas (Figure 1C).

### 2.2. Ectopic Notch3 Signaling Activation Compromises Renal Function and Increases Kidney Size

**Figure 2:**
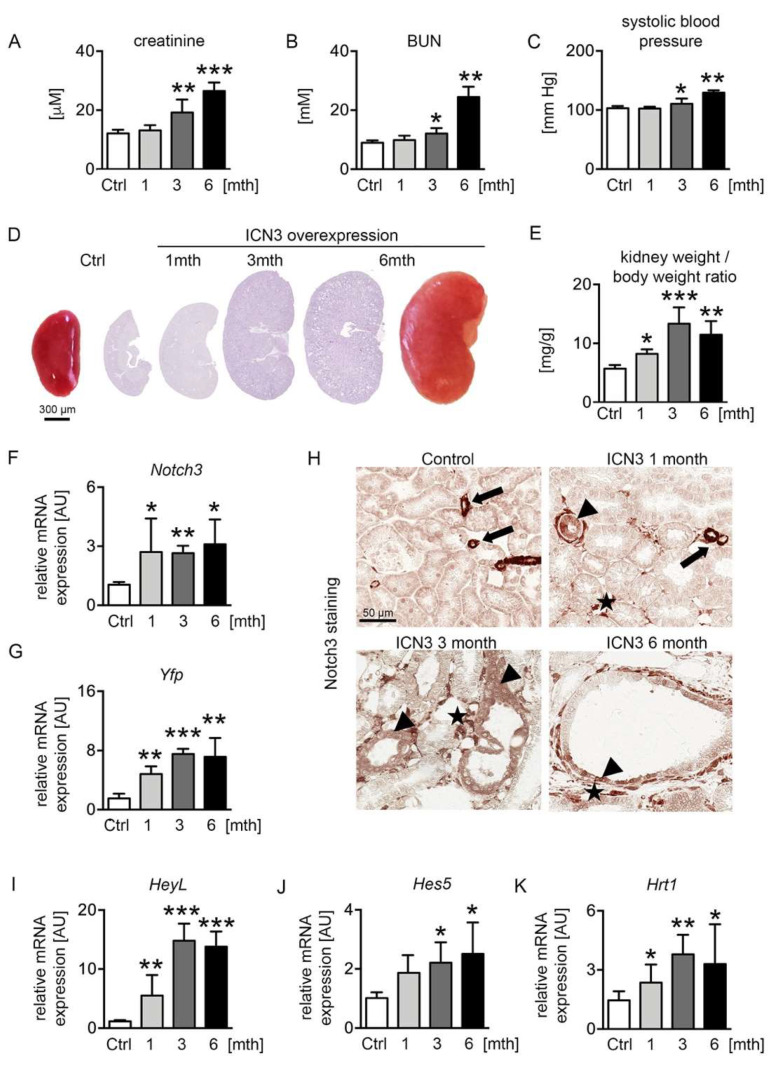
Notch3 overexpression alters renal function and structure. Two-month-old male N3ICD-overexpressing mice were treated for 10 days with doxycycline (dox) in the drinking water to activate N3ICD expression. Renal function was analyzed after one month (1mth, n = 5), three months (3mth, n = 6) and six months (6mth, n = 4) of overexpression and compared to control mice not treated with doxycycline (Ctrl, n = 5). Plasma creatinine (**A**), BUN (**B**) and systolic blood pressure (**C**) were significantly increased after chronic N3ICD overexpression in tubular epithelial cells. Afterwards, mice were sacrificed, and the kidney analyzed. Macro- and microscopic (PAS stain) analysis (**D**) and kidney per body weight ratio (**E**) showed a massive increase in kidney size after three months. N3ICD overexpression was confirmed by real-time PCR (**F**) and immunohistochemistry for Notch3 (**H**), and the gene expression of the Notch3 tag *Yfp (yellow fluorescent protein*) (**G**) and classical Notch target genes, i.e., *HeyL* (**I**), *Hes5* (**J**) and *Hrt1* (**K**). * *p* < 0.05; ** *p* < 0.01; *** *p* < 0.001, compared to control.

We have previously generated transgenic mice overexpressing the Notch3 intracellular domain (N3ICD) in tubular epithelial cells [9]. To examine the effect of Notch3 activation on the tubular epithelial cell phenotype, two-month-old male mice were treated with doxycycline (dox) in the drinking water for ten days to activate N3ICD expression. Mice were sacrificed after one, three and six months of overexpression (abbreviated 1mth, 3mth and 6mth, respectively). We measured renal functional parameters, i.e., serum creatinine and blood urea nitrogen (BUN), and systolic blood pressure. Serum creatinine and BUN levels were normal after the first month of doxycycline treatment but progressively increased at the later time points, demonstrating the significant deterioration of renal function after chronic N3ICD overexpression (Figure 2A,B). Similarly, systolic blood pressure increased over time (Figure 2C). Macroscopic and microscopic examination of N3ICD kidneys revealed a significant enlargement (Figure 2D,E).

Notch3 expression was monitored with real-time PCR for *Notch3* and *Yfp* (yellow fluorescent protein) expression, the latter tagging the N3ICD-overexpressing cassette (Figure 2F,G). Stable overexpression of N3ICD was found during the 6 months. Immunohistochemistry for N3ICD showed the expected vascular expression (Figure 2H arrows) in control mice. After doxycycline administration, N3ICD was expressed in tubules (Figure 2H arrowheads) and interstitial cells (Figure 2H stars). To verify that the transgene could activate the Notch signaling pathway, the expression of several downstream targets of Notch was evaluated. *HeyL* showed the most prominent increase from early on, and *Hes5* and *Hrt1* were also significantly upregulated (Figure 2I–K).

Collectively, renal epithelial activation of Notch3 signaling resulted in significant kidney enlargement and deterioration of renal function.

### 2.3. Notch3 Activation Generates Heterogeneous Cyst Formation

**Figure 3:**
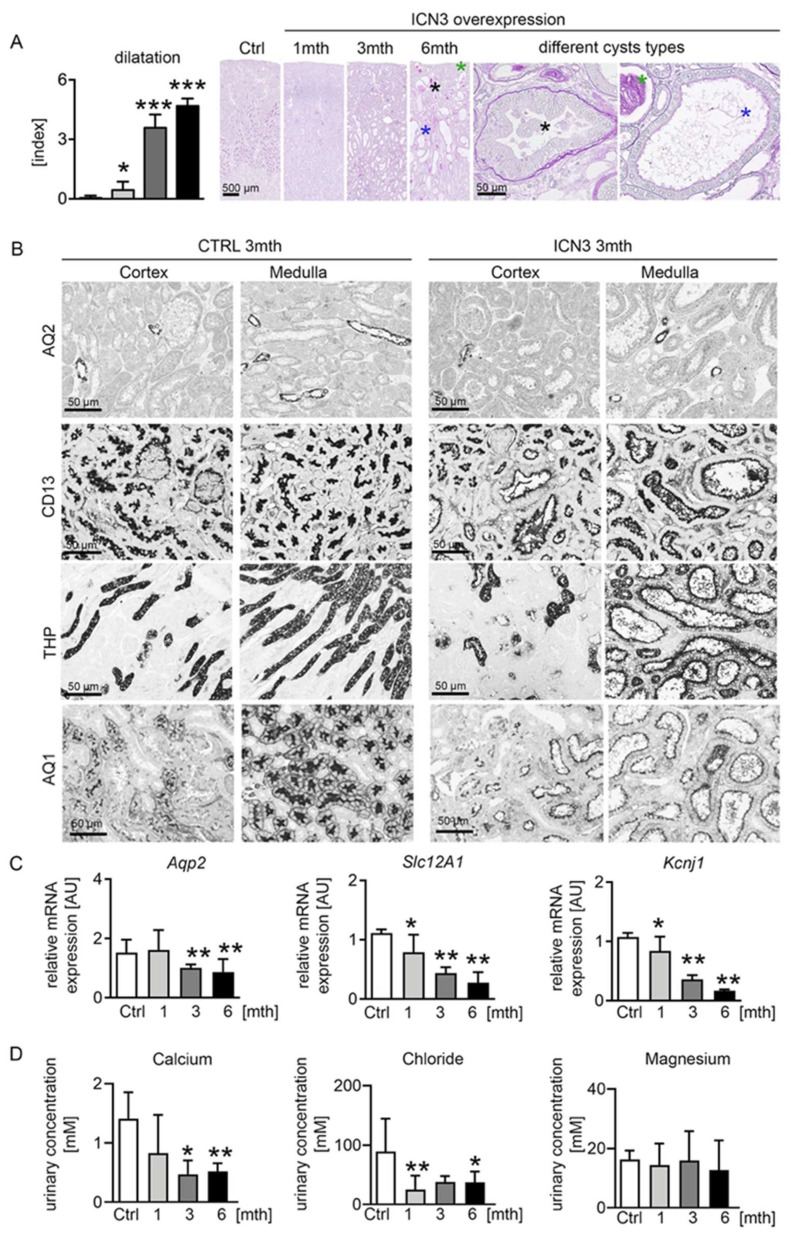
Tubular phenotype after N3ICD overexpression. Scoring of tubular dilation in PAS staining revealed a significant increase and different cyst types (**A**). Highly dilated tubules with monolayer of cuboid tubular epithelial cells that maintained their brush border are marked with a blue asterisk; highly dilated tubules filled with PAS-positive stained material, consisting of flattened cells that had no brush border, are marked with a green asterisk; and tubules showing hyperplastic lesions with multilayered, columnar epithelial cells with protrusions into the cystic lumen are marked with a black asterisk. Immunohistochemistry for markers of different tubular segments, i.e., aquaporin 2 (AQP2) for collecting ducts, CD13 for proximal tubules, Tamm–Horsfall protein (THP) for the loop of Henle and distal tubules and aquaporin 1 (AQ1) for proximal tubules and descending loop of Henle, showed that proximal and distal tubules were largely affected three months after doxycyclin treatment (**B**). Real-time PCR for the collecting duct water channel *aquaporin 2* showed a decrease after 3mth, whereas the potassium channel ROMK (*Kcnj1*) and the multi-ion transporter NKCC2 (*Slc12A1*), localizing in the loop of Henle, decreased progressively starting from 1mth (**C**). Urinary calcium and chloride concentrations were significantly decreased, whereas the magnesium concentration was not affected (**D**). * *p* < 0.05; ** *p* < 0.01; *** *p* < 0.001, compared to control.

Histological examination demonstrated that morphologic changes started in the outer stripe of the outer medulla (OSOM). In control kidneys, the border between the cortex and medulla was clearly visible. Already one month after doxycycline treatment, tubules, especially in the OSOM, were bigger, hyperplastic and slightly dilated (Figure 3A). Over time, the tubular dilation increased towards cyst formation, and an increasing proportion of tubules in both the OSOM and renal cortex became affected (Figure 3A). The tubular cysts varied in size and appearance, identifying three main types of cyst phenotypes: (1) Cystic tubules with a monolayer of cuboid tubular epithelial cells that maintained their brush border. They occurred early, originally in the OSOM and only later in the cortex, and were hyperplastic, suggesting high proliferation (Figure 3A blue asterisk). (2) Cystic tubules filled with PAS-positive stained material and flattened tubular epithelial cells that had no brush border (Figure 3A green asterisk), occurring only at the latest time point. (3) Tubules showing hyperplastic lesions with multilayered, columnar epithelial cells with protrusions into the cystic lumen (Figure 3A black asterisk), also observed only at later stages. A common feature in all these cysts was a thickened basement membrane, especially in the hyperplastic cysts. The hyperplastic lesions showed no clear cells and no papillary growth. Additionally, no (micro)invasive growth was found, and no tumor formation was observed, at any time point. Additionally, macroscopically, no metastases were found in other organs. To identify the tubular segment involved in cyst formation, we stained for different tubular segment markers, i.e., aquaporin 2 (AQP2) for collecting ducts, CD13 and aquaporin 1 (AQP1) for proximal tubules and Tamm–Horsfall protein (THP) for the thick ascending limb (TAL). Proximal, distal and TAL cells appeared to equally contribute to the pool of dilated tubules and cysts, while collecting ducts retained their shape (Figure 3B). Next, we performed real-time PCR for several epithelial ion transporters that can serve as markers of normal tubular function. Transcription levels of the medullary potassium channel ROMK (Kcnj1) and the multi-ion transporter NKCC2 (Slc12A1), localizing in the loop of Henle, decreased progressively starting from one month, leading to a decrease in the ion concentration in the urine (Figure 3C). The collecting duct water channel aquaporin 2 decreased after three months (Figure 3B,C).

Collectively, Notch3 overexpression induced distinct pathological cystic tubular alterations leading to a deterioration of tubular function with decreased ion channel expression, consistent with the development of cystic kidney disease.

### 2.4. Epithelial N3ICD Induces Severe Tubular Damage and cCll Dedifferentiation

**Figure 4:**
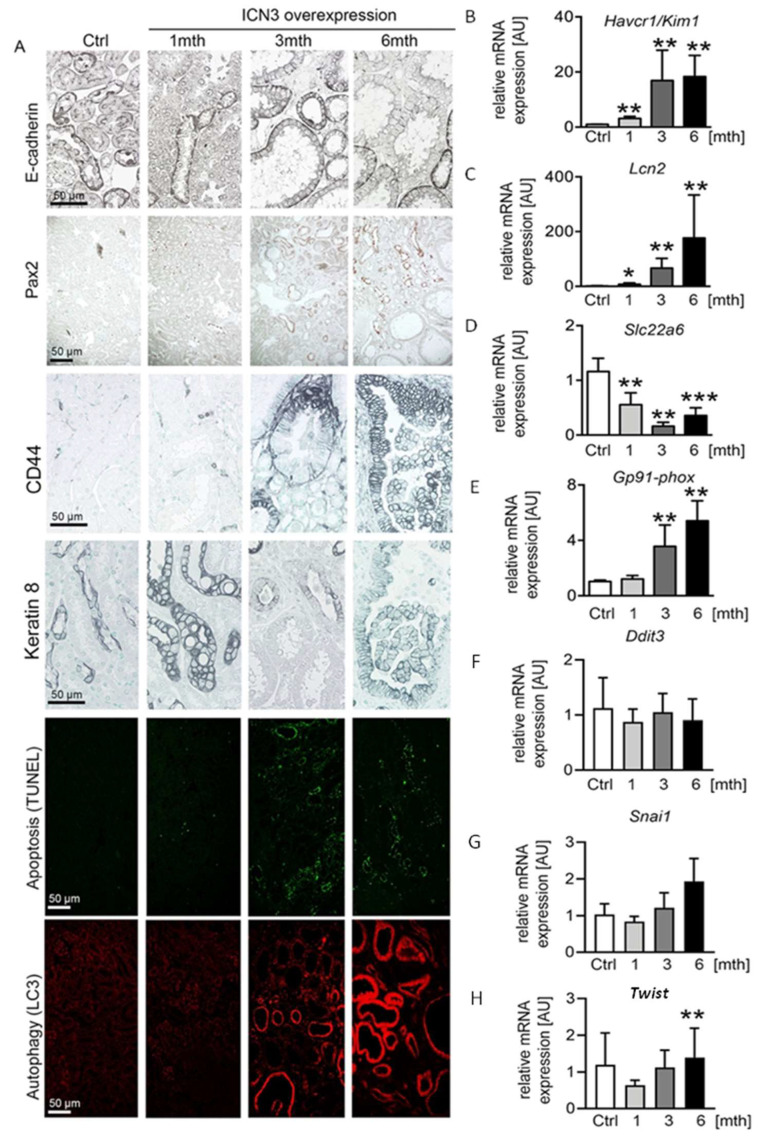
Notch3 induces severe tubular epithelial cell stress and apoptosis. E-cadherin immunostaining decreased with time in response to Notch3 signaling activation (**A**). In addition, developmental transcription factor Pax2 was re-expressed in adult cortical tubular cells after N3ICD overexpression and increased with time (**A**), suggesting a dedifferentiation of the tubules. Apoptosis evaluation with TUNEL and autophagy evaluation with LC3 immunostaining revealed increased tubular cell death (**A**). Immunohistochemistry of tubular injury markers CD44 and keratin 8 (**A**), as well as real-time PCR for *Havcr1/Kim1* (**B**) and *Lcn2/Ngal* (**C**) and for oxidative stress markers *Slc22a6* (**D**) and *GP91phox* (**E**), revealed tubular injury already after one month. *Ddit3* (CHOP) (**F**) and *Snai1* (Snail) (**G**) were not changed due to N3ICD overexpression, whereas *Twist* significantly increased at 6 months (**H**). * *p* < 0.05; ** *p* < 0.01; *** *p* < 0.001, compared to control.

The water channels aquaporin 1 and 2 showed normal apical localization in the doxycycline-treated mice, suggesting that that Notch3 activation did not influence cell polarity (Figure 3B). Additionally, E-cadherin expression showed a similar localization, but expression was reduced over time in N3ICD mice (Figure 4A). Since decreased E-cadherin expression is a marker of tubular epithelial cell dedifferentiation [11,12], we analyzed the expression of Pax2, a transcription factor critical during renal development. It is mainly expressed in medullary tubular epithelial cells in the adult kidney [13] and in several renal cell carcinomas and is considered a marker of dedifferentiation [14]. The renal cortex of control mice was negative for Pax2. After one month of Notch3 activation, Pax2 was observed in a few tubular epithelial cells (Figure 4A). At three and six months, a significant number of cortical tubular epithelial cells expressed Pax2 (Figure 4A). Interestingly, strongly enlarged tubules were Pax2 negative, while less strongly dilated tubules were positive, indicating re-activation of Pax2 expression during the process of tubular enlargement.

Next, we analyzed the phenotype of tubular epithelial cells using CD44 and Havcr1/Kim1, markers for tubular injury and regeneration. In healthy mice and mice one month after doxycycline treatment, CD44 was expressed only by some immune cells. At the later stages, CD44 was de novo expressed by tubular epithelial cells, increased over time and found in each cyst type and independently of the cell shape (flattened, cuboid or columnar) (Figure 4A). In line with the increased CD44 expression, *Havcr1/Kim1* mRNA expression increased significantly over time (Figure 4B).

To differentiate between tubular epithelial cell injury and regeneration, we analyzed stress and injury markers. We measured the expression of the cytoskeleton filament keratin 8, whose overabundance is a feature in ADPKD. Keratin 8 was only expressed by the collecting duct epithelium in healthy mice. After N3ICD activation, keratin 8 expression in collecting duct cells increased and was de novo expressed in dilated tubules (Figure 4A). *Lcn2/NGAL*, a widely used marker of tubular stress and injury, showed a similar expression pattern to *Havcr1/Kim1*, with a small increase at one month being followed by an important upregulation after three months (Figure 4C).

To assess whether the increased tubular injury is associated with autophagy and cell death, we evaluated apoptosis with the TUNEL assay and autophagy with LC3 staining. TUNEL revealed a very limited number of apoptotic epithelial cells one month after N3ICD induction that highly increased after three and six months (Figure 4A). Apoptotic cells were usually absent from strongly enlarged tubules, possibly indicating an escape from cell regulatory mechanisms. LC3 staining revealed a strong increase after three months (Figure 4A). By six months, all highly dilated tubules were positive for LC3 (Figure 4A). To examine whether additional mechanisms contributed to epithelial stress and death, we measured the expression of markers of oxidative and endoplasmic reticulum stress (Figure 4D–H). A decrease in OAT1 is considered as a marker of oxidative stress [15]. Transcripts of organ anion transporter 1 (OAT1/*Slcc26*) of the proximal tubules significantly decreased from one month (Figure 4D). To verify that N3ICD overexpression induced oxidative stress, we also quantified the expression of the NADPH subunit GP91 phox. Starting from three months of N3ICD, expression of *Gp91-phox* significantly increased, supporting the involvement of oxidative stress in this model (Figure 4E). In contrast, gene expression of transcription factors involved in endoplasmic reticulum stress (*Chop/Ddit3*) or epithelial-to-mesenchymal transition and tumor progression (*Twist* and *Snail*) were not significantly altered (Figure 4F–H).

Overall, Notch3 activation in adult tubular cells resulted in severe phenotypic alterations characterized by epithelial injury, dedifferentiation and cell death.

### 2.5. Notch3 Activation Leads to Hyperplasia and Disturbed Alignment of Cell Division

**Figure 5:**
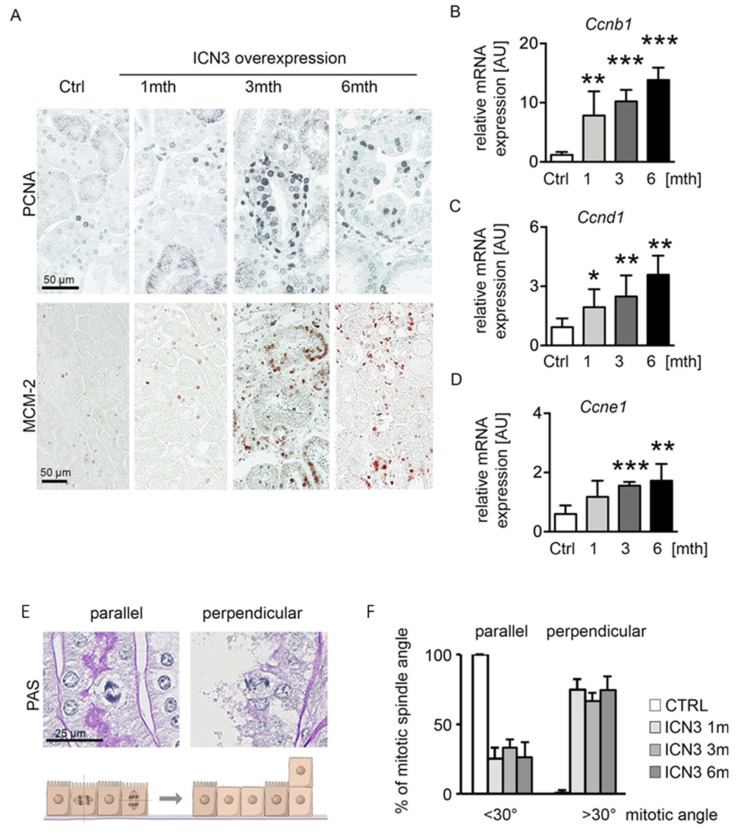
N3ICD overexpression induces proliferation. Immunohistochemistry for proliferation markers PCNA and MCM-2 (**A**) revealed increased tubular proliferation in response to N3ICD signaling. Quantification of cyclin b (*Ccnb1*; (**B**)), cyclin d (*Ccnd1*; (**C**)) and cyclin E (*Ccne*; (**D**)) mRNA expressions confirmed that proliferation increased in a time-dependent manner. In addition, Notch3 overexpression altered mitotic spindle orientation from planar to perpendicular (**E**,**F**). * *p* < 0.05, ** *p* < 0.01; *** *p* < 0.001, compared to control.

PKD is characterized by increased epithelial proliferation. To evaluate Notch3’s effect on proliferation and cyst formation, we measured proliferation using PCNA and MCM-2 (Figure 5A). A slight increase in proliferating cells one month after Notch3 activation was found, which peaked after three and slightly decreased after six months. To quantify proliferation at the different time points, we assessed cyclin expression with real-time PCR. *Ccnb1*, *Ccnd1* and *Ccnde1* showed identical expression patterns, with a time-dependent progressive increase (Figure 5B–D).

The hyperplastic multilayered epithelium in several tubules suggests disorganized mitoses. Therefore, we quantified the mitotic spindle orientation and the occurrence of perpendicular proliferation (Figure 5E,F). Mitotic spindle angles <30° were defined as a mitotic spindle parallel to the basement membrane, while angles >30° were defined as a perpendicular orientation that can lead to cell division on top of each other (Figure 5E). Control mice showed a parallel mitotic spindle orientation, whereas N3ICD-overexpressing mice exhibited a reduced parallel and highly increased perpendicular orientation (Figure 5F).

These data suggest that overexpression of N3ICD induced proliferation with a disoriented mitotic spindle.

### 2.6. Epithelial N3ICD Resulted in Tubulointerstitial Inflammation and Fibrosis

**Figure 6:**
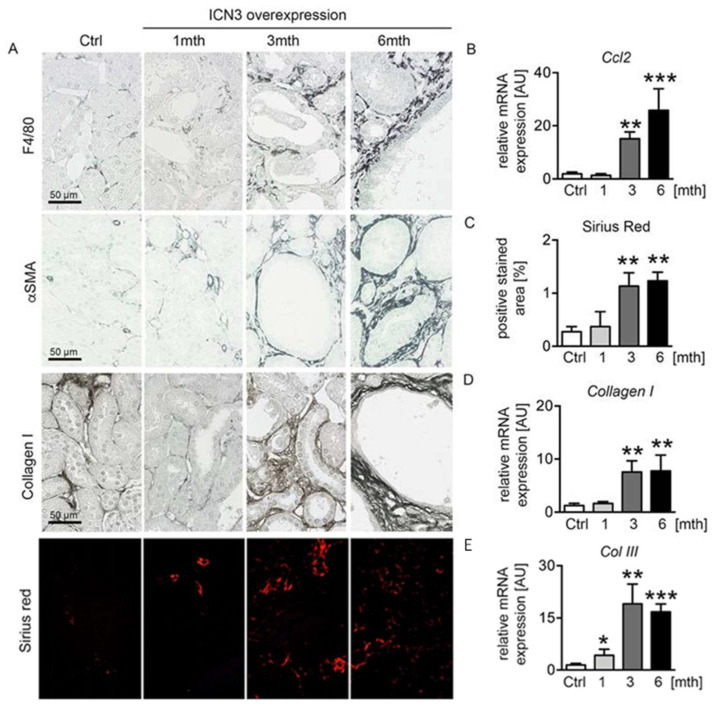
Notch3 overexpression results in severe tubulointerstitial inflammation and fibrosis. F4/80 immunostaining was used for evaluation of infiltrating macrophages, α-smooth muscle actin (αSMA) for fibroblast activation and collagen I and Sirius red for abnormal matrix deposition (**A**). Measurements of mRNA expressions of *Ccl2* (**B**), *Col I* (**D**) and *Col III* (**E**) and quantification of Sirius red staining (**C**) confirmed increased inflammation and fibrosis in mice overexpressing N3ICD in a time-dependent manner. * *p* < 0.05; ** *p* < 0.01; *** *p* < 0.001, compared to control mice.

Polycystic kidney disease is characterized by renal fibrosis, and the extent of fibrosis might be an important sign of PKD progression [16]. We have previously reported that epithelial cells expressing Notch3 promote an inflammatory response and activate infiltrating macrophages [7]. Chronic epithelial overexpression of N3ICD resulted in severe tubulointerstitial inflammation and fibrosis that progressed with time. Immunohistochemistry for the macrophage marker F4/80 showed a small number of infiltrating macrophages at one month that significantly increased at three months and six months, exhibiting peritubular localization (Figure 6A). Real-time PCR for *Ccl2* confirmed the time-dependent evolution of inflammation (Figure 6B). Accordingly, interstitial fibrosis assessed with immunohistochemistry for α-smooth muscle actin (αSMA) and collagen I, and Sirius red staining (Figure 6A,C) progressed in parallel with inflammation. Real-time PCR for collagens I and III (Figure 6D,E) confirmed the fibrotic response.

## 3. Discussion

We studied the impact of Notch3 expression in the renal tubular epithelium. A main finding was a significant upregulation of Notch3 in epithelial cells of all PKD cases and renal cell carcinomas. Another major result was that activation of Notch3 in tubular epithelial cells resulted in cystic kidney disease and pre-neoplastic lesions. We found that Notch3 signaling regulates cell proliferation and restricts the orientation of epithelial cell division relative to the tubular basement membrane. Our study proposes Notch3 as a new and important player in the progression of human tubular epithelium-originated diseases.

Cystic kidney disease can be acquired over time in patients with chronic kidney disease and is more likely in patients who are on dialysis. In addition to acquired cystic kidney disease, renal cystic diseases can occur due to genetic mutation. Autosomal dominant polycystic kidney disease is among the most common, life-threatening genetic disorders, affecting 1:500 adults worldwide [17]. ADPKD is caused by mutations in the genes encoding for Polycystin-1 and -2, PKD-1 and PKD-2, respectively. Idowu et al. showed that Notch pathway components were consistently upregulated or activated in cyst lining cells in mouse models of ADPKD and ARPKD and in ADPKD patients. Of interest, the activated intracellular domain of Notch3 was detectable and expressed predominately in the proliferating cystic epithelium [18]. This is in line with our results showing a de novo expression of Notch3 in dilated tubules and in the multilayered epithelium of patients with ADPKD. We also found a similar increase in Notch3 in patients with ACKD, suggesting a common mechanism that activates Notch3 during cyst formation.

We hypothesized that Notch3 plays a crucial role in cyst formation. Therefore, we overexpressed the active intracellular domain of Notch3 in tubular epithelial cells and analyzed the mice at different times after doxycycline treatment, i.e., one, three and six months later. It has already been described that overexpression of the intracellular domain of Notch1 in tubular epithelial cells leads to increased tubular cell proliferation and interstitial fibrosis via controlled cell proliferation with some degree of dysplasia, but not to cysts [19]. Comparable to the overexpression of Notch1, we found an increased proliferation of tubular epithelial cells already one month after doxycycline treatment. Tubules already increased in diameter, but the dilation was moderate and similar to published data of Notch1 overexpression. After three months, and even more after six months, of Notch3 activation, tubular dilatation increased remarkably and renal tubular cysts emerged. This difference between Notch1 and Notch3 overexpression can be explained by the different time points of analysis. Notch1-overexpressing mice were analyzed 4 weeks after induction because the mice appeared sick and died at approximately 5 weeks after initiation. The mechanism that leads to premature death has not been described, but this suggests that Notch1 induces some additional mechanisms.

Our results are further in line with studies of the zebrafish double bubble (dbb), indicating that inhibition of Jagged2 or of Notch3 reduced cyst formation [20,21]. Knock-down of Jagged2 and Notch3 increased expression of the ciliary gene *rfx*. Inhibition of Notch signaling, either by administrating a γ-secretase inhibitor or by knock-down of Jagged2, rescued cyst formation in dbb mutant zebrafish. These studies indicate that increased Notch signaling causes ciliary dysfunction, leading to renal cysts.

Interestingly, downregulation of Notch signaling in the kidney mesenchyme in mice also caused tubular cysts, with severity corresponding to the degree of inhibition of the Notch pathway [22]. Genetic deletion of Notch1 or Notch2 resulted in renal cysts by three weeks of age, but ablation of Notch1 or Notch2 simultaneously, or of RBP-J, resulted in renal cysts at birth [22]. It is unknown whether downregulation of Notch1 or Notch2 after birth would also induce tubular cysts or if these are developmental effects. In contrast to Notch3-deficient mice, which show no serious development defects, Notch1 and Notch2 deficiency is embryonically lethal, further confirming a more vital role for Notch1 during embryonic development compared to Notch3.

In mice lacking Notch2 and one allele of Notch1, the mitotic spindle orientation of epithelial cells of pre-cystic proximal tubules was disrupted relative to the basement membrane [22]. This was proposed to result in epithelial stratification. We also observed an altered spindle orientation in Notch3-overexpressing mice, which resulted in a multilayered epithelium and protrusions into the cystic lumen in some of the renal cysts. Our study reveals that Notch3 signaling is required for maintaining the alignment of a cell division plane perpendicular to the basement membrane in renal epithelial cells. Most of the epithelial cells lining the cysts remain in a monolayer and result from hyperproliferation of the tubular epithelial cells. We conclude that an impaired spindle orientation coupled with increased numbers of mitotic events allows some cells to stratify. This cellular defect does not commonly occur in PKD rodent models [23]. Still, these data suggest that mitotic spindle orientation defects, whether they are relative to the tubular axis or to the tubular basement membrane, may be a plausible mechanism in the pathogenesis of renal cysts. It is described that ciliopathies affecting the position of the centrosome and hence the orientation of the mitotic spindles during cell division lead to dysregulation of the tubular diameter and cyst formation [24]. It is likely that maintaining the tubular epithelium of the nephron as a monolayer requires that the mitotic spindles be restricted parallel to the basement membrane.

Notch signaling is required for maintenance of mature renal tubular epithelial cells [25,26]. We found that activation of Notch3 had no influence on cell polarity but seemed to induce cell dedifferentiation and encourage hyperplasia. Although most cysts are lined by a single layer of epithelium, hyperplastic lesions are occasionally seen in which the epithelial cells line cysts in multilayer or even papillary protrusions.

Emerging evidence has shown that deregulated expression of Notch receptors, ligands and targets is observed in many types of tumors [22,27,28]. Notch may play a role in tumor genesis by inhibiting differentiation, promoting survival or accelerating proliferation due to its capacity to induce expression of the myc oncogene [29,30]. Notch1, the most studied member of the family, was shown to induce tubular hyperplasia when overexpressed in tubular epithelial cells [19], and Notch1 and Jagged1 are overexpressed in human clear cell renal cell carcinoma (ccRCC) compared to normal kidneys. Furthermore, previous experimental studies reported that Notch3 and Jag1 mRNA is elevated in ccRCC [27,28], and transcriptomic analysis of clear cell RCC tissue revealed upregulation of Notch1, Notch3 and several members of the Notch signaling pathway [31]. Whole-exome sequencing of patients with tuberous sclerosis complex-associated renal cell carcinoma and microarrays in different types of renal tumors also revealed increased Notch3 expression in neoplastic tissue [27,28]. Overexpression of Notch1 in mice with mutated VHL led to the development of renal cell carcinoma, structurally resembling the human pathology. Inversely, blocking Notch signaling with γ-secretase inhibitor treatment attenuated neoplastic cell formation [31]. These data link Notch signaling with renal cell carcinoma and suggest its role in tumor genesis. This led us to analyze the expression of Notch3 in major renal cell carcinoma subtypes and to reanalyze available public data. Our results show an increased upregulation of Notch3 in various renal cell carcinomas. On the other hand, activation of Notch3 did not induce renal cell carcinoma in our animal model, suggesting either a second hit or longer duration of activation is necessary for the development of RCC.

Our results illustrate that Notch3 activation can be involved in the development and formation of cysts. Therefore, mutations of Nocth3 that lead to a gain of function and thus to a constitutive activation of Notch signaling may cause cystic kidney diseases in humans, whether acquired during kidney aging or congenital with a later expression in life. In order to investigate this possibility, it will be interesting to scan for such mutation patients with chronic kidney disease and numerous bilateral cysts.

In conclusion, we found that activation of Notch3 in the renal tubular epithelium leads, over time, to the formation of renal cysts and the concomitant decline in renal function. This pathological process occurs because aberrant Notch3 signaling influences proliferation, the alignment of cell division and dedifferentiation in the proximal and distal tubules, thus favoring the progression of cystic renal diseases and eventually the development of renal cell carcinoma.

## 4. Materials and Methods

### 4.1. Pax8-rtTA-LC1-R26-N3ICSAT (N3ICD) Mice

The generation of N3ICD-overexpressing mice in TECs has been previously described [9]. N3ICD expression was induced by administration of 0.2 mg/mL dox in the drinking water, containing 3% sucrose, for a period of 10 days, always starting one month after birth. Mice were sacrificed after 1, 2, 3 and 6 months after dox administration, n = 6 per time point. Control mice were administered 3% glucose in the drinking water for 10 days and sacrificed at the respective time points (n = 6 per time point). In a separate study, four mice per time point were used for systolic blood pressure measurements. The left femoral artery was catheterized for measurement of arterial pressure via a pressure transducer (Statham P23 DB) as described elsewhere [32]. In total, 80 transgenic mice were used. All mice were handled in strict accordance with good animal practice as defined by the relevant national animal welfare bodies of France, and all animal work was approved by the appropriate committee of the National Institute for Health and Medical Research (INSERM) and the Sorbonne University (Paris, France). Animals were housed at a constant temperature with access to water and food ad libitum.

### 4.2. Histological and Functional Parameters

Half kidneys from each animal were fixed in 4% formalin solution and embedded in paraffin. Then, 3 μm sections were stained with Masson’s trichrome and PAS for histological evaluation of N3ICD mice. Tubular dilation and necrosis were evaluated semi-quantitatively, using the following scale: 0, no tubular damage; 1, damage in 1–25% of the tubules analyzed; 2, damage in 26–50% of the tubules analyzed; 3, damage in 51–75% of the tubules analyzed; 4, damage in >76% of the tubules analyzed. Scoring was performed in a masked manner on coded slides by two different investigators. Interstitial fibrosis was determined semi-quantitatively on Sirius red-stained paraffin sections at a magnification of ×200 using computer-based morphometric analysis software (Analysis, Olympus, Rungis, France). BUN and creatininemia levels were measured with an enzymatic method (Konelab automater, Illkirsch, France) and expressed in mM and μM, respectively.

### 4.3. Human Renal Tissue

Human renal tissue was obtained from nephrectomy specimens with renal cell carcinoma or polycystic kidney disease taken from the archives of the Institute of Pathology RWTH Aachen. Control kidney tissues were taken either from tumor-free tissue from RCC nephrectomies or from patients who underwent nephrectomy due to trauma, both without other known renal diseases and obvious histopathological alterations. All human samples were handled anonymously and analyzed in a retrospective manner, and the study was approved by the local review board (EK244/14 and EK042/17) and in line with the Declaration of Helsinki of 1975, as revised in 2000 and 2013.

### 4.4. Total RNA Extraction and Quantitative Real-Time PCR

Total RNA was extracted from renal tissue using TRIzol reagent (Invitrogen, Waltham, MA, USA). RNA quality was checked by control of the optical density at 260 and 280 nm. Total RNA from cells was extracted using Spin Column Total RNA Mini-Preps Super Kit (Proteogenix, Schiltigheim, France) according to the manufacturer’s instructions. Contaminating genomic DNA was removed by RNase-free DNAse (Qiagen, Hilden, Germany) for 30 min at 37 °C. cDNA was synthesized from 1 µg of purified RNA using oligo-dT and superscript II RT (Qiagen) for 1 h 30 min at 37 °C and 10 min at 70 °C. qPCR experiments were performed as previously described [9]. Analysis of relative gene expression was conducted by using the 2^−ΔΔCT^ method. Results are expressed as the ratio of the target gene/internal control gene (HPRT). Sequences of primers used in our studies are listed in Table 1.

### 4.5. Immunostaining

Immunohistochemistry was performed on 3 μm-thick paraffin-embedded tissue sections. Tissue was de-paraffinized, and 10 mM citric acid, pH 6, at 95 °C, was used for antigen retrieval. Sections were permeabilized with 0.1% triton/PBS. Antibodies against Notch3 (Abcam, Cambridge, UK, ab23426), F4-80 (AbD Serotec, Oxford, UK, MCA497R), MCM2 (Cell Signaling, Danvers, MA, USA, #4007), pax2 (Abcam, ab150391), PCNA (Merck, Darmstadt, Germany, #NA03), aquaporin 1 (Alpha diagnostic AQ11-A), aquaporin 2 (Abcam, ab15116), CD13 (Abcam, ab 108310) and Tamm–Horsfall protein (Santa Cruz, Dallas, TX, USA, sc-20631) were used. Immunofluorescence for LC-3 (Novus Bio, Littleton, CO, USA, 100-2220) was performed on 3 μm-thick paraffin-embedded tissue in a similar manner. Alexa fluor (Invitrogen) secondary antibody was used for detection. Images were obtained with an OlympusIX83 photonic microscope at ×200 magnification.

### 4.6. TUNEL Assay

Apoptosis was evaluated with the DeadEndTM Fluorometric TUNEL System (Promega, Madison, WI, USA) according to the manufacturer’s instructions on 3 μm paraffin sections. Quantification was performed by expressing the number of TUNEL+ tubules in the outer and inner cortices per total number of tubules in the same areas. TUNEL+ tubules were considered tubules with at least one TUNEL+ epithelial cell.

## Figures and Tables

**Table 1:** Primers for real-time PCR (all for murine genes).

Gene	Primer Sequence
Notch3	FW: CCCCAACCAGAAGTTACCCC RV: AGGAGTGTCACTTCAGCACC
HeyL	FW: CTGAATTGCGACGATTGGT RV: CTGAATTGCGACGATTGGT
Hrt1	FW: CATGAAGAGAGCTCACCCAGA RV: GAACACAGAGCCGAACTCAA
Hes5	FW: CCCAAGGAGAAAAACCGACT RV: TGCTCTATGCTGCTGTTGATG
Yfp	FW: CTCGTGACCACCTTCGGCT RV: TCCTGGACGTAGCCTTCG
Havcr1/Kim1	FW: TCAGATTCAAGTCTTCATTTCAGG RV: CCCCCTTTACTTCCACATAAGAA
Lcn2 (NGAL)	FW: CCATCTATGAGCTACAAGAGAACAAT RV: TCTGATCCAGTAGCGACAGC
Slc12A1 (NKCC2)	FW: ATGCCTCGTATGCCAAATCT RV: CCCACATGTTGTAAATCCCATA
Kcnj1 (ROMK)	FW: GGTGCAAGGGACTTTCTCAC RV: TGAACATCCTTTCTGTCAGTGC
Aqp2	FW: TAGCCCTGCTCTCTCCATTG RV: GAGCAGCCGGTGAAATAGAT
Ccnb1	FW: TCTTCTCGAATCGGGGAAC RV: TTGGCCTTATTTTCTGCGTTA
Ccnd1	FW: CATCCATGCGGAAAATCG RV: CAGGCGGCTCTTCTTCAA
Ccne1	FW: ACGGGTGAGGTGCTGATG RV: GGACGCACAGGTCTAGAAGC
Ccl2 (MCP-1)	FW: CATCCACGTGTTGGCTCA RV: CATCCACGTGTTGGCTCA
Collagen I	FW: GCAGGTTCACCTACTCTGTCCT RV: CTTGCCCCATTCATTTGTCT
Collagen III	FW: TGGTTTCTTCTCACCCTTCTTC RV: TGCATCCCAATTCATCTACGT
GP91phox	FW: ACTGCGGAGAGTTTGGAAGA RV: GGTGATGACCACCTTTTGCT
Slc22a6 (OAT-1)	FW: CCTATGCTGTGCCCCACT RV: GGCTGACTCAATGAAGAACCA
Hprt	FW: GGAGCGGTAGCACCTCCT RV: CTGGTTCATCATCGCTAATCAC
Ddit3 (CHOP)	FW: GCGACAGAGCCAGAATAACA RV: GATGCACTTCCTTCTGGAACA
Snai1 (Snail homologue 1)	FW: GTCTGCACGACCTGTGGAA RV: CAGGAGAATGGCTTCTCACC
Twist	FW: AGCTACGCCTTCTCCGTCT RV: TCCTTCTCTGGAAACAATGACA

## Data Availability

Not applicable.
